# Long-term fertilization alters soil properties and fungal community composition in fluvo-aquic soil of the North China Plain

**DOI:** 10.1038/s41598-020-64227-6

**Published:** 2020-04-29

**Authors:** Yan-Chen Wen, Hai-Yan Li, Zhi-An Lin, Bing-Qiang Zhao, Zhan-Bin Sun, Liang Yuan, Jiu-Kai Xu, Yan-Qing Li

**Affiliations:** 1 Key Laboratory of Plant Nutrition and Fertilizer, Ministry of Agriculture, Institute of Agricultural Resources and Regional Planning, Chinese Academy of Agricultural Sciences, Beijing, 100081 P.R. China; 2 School of Light Industry, Beijing Technology and Business University, Beijing, 100048 P.R. China

**Keywords:** Microbiome, Fungal ecology

## Abstract

Different fertilization regimes can substantially influence soil fungal community composition, yet fewer studies try to control for the effects of nitrogen input. Here, we investigated the impact of fertilization with equal nitrogen upon soil properties and soil fungal diversity and community composition in the North China Plain in a long-term field experiment. Long-term (32 years) fertilization regimes were applied with equal amounts of nitrogen: no chemical fertilizer or organic manure; chemical fertilization only; organic manure fertilization only, and; combination of 1/2 chemical fertilizer and 1/2 organic manure. Then we investigated the influence of these four fertilization regimes to soil properties, fungal diversity and community composition. The results showed that applying organic manure significantly influenced soil properties. Illumina MiSeq sequencing and its analysis revealed that organic manure fertilization significantly changed soil fungal alpha diversity, but chemical fertilization did not. Although soil fungal community composition did not differ significantly among all the fertilization regimes at the phylum and class levels, they did show differences in the abundance of dominant fungi. Yet at the genus level, soil fungal community composition, abundance, and beta diversity was affected by all fertilization regimes. Application of organic manure also reduced the abundance of soil-born fungal pathogens such as *Fusarium*. Our results suggest that long-term application of organic manure could markedly improve soil properties, altering soil fungal community composition and its diversity. Moreover, organic manure fertilization could limit soil-born fungal diseases, to further contribute to soil ecosystem sustainability.

## Introduction

The North China Plain is the most important crop production area in China, being a typical region where winter wheat and summer maize are planted within the same year. Their respective yields there account for more than 50% and 30% China’s national totals^1,2^. The Plain has many soil types, including brown soil^3^, cinnamon soil^4^, seashore saline soil^5^, and fluvo-aquic soil^6^, but the last is the most wildly distributed and most used for winter wheat–summer maize rotations in the Plain.

Fertilization is an effective way to increase crop yield and improve soil fertility. The use of fertilization regimes may be divided into three types according to fertilizer category: chemical fertilizer, organic manure, and their combination^7–9^. Applying chemical fertilizer can ensure a relatively high crop yield; however, this fertilization regime is no longer suitable because of its low resource utilization rate, weak sustainability, and the severe adverse impact it has on the environment^10,11^. By contrast, using organic manure has environment-friendly effects, but the associated crop yield generally remains too low few to sustainably meet the food demands of an increasing human population^12,13^. Therefore, using neither chemical fertilizer nor organic manure fertilization seem viable options for ensuring crop production. However, the combination of organic manure with chemical fertilizer may produce a higher crop yield, resource utilization rate, and environmental benefits compared with single fertilization of chemical fertilizer or organic manure^14,15^. To date, most reports testing these three fertilization regimes were conducted as short-term experiments, which could, to some extent, be influenced by stochastic environmental factors that lead to weakly representative findings. Long-term field experimentation is more robust scientifically for inferring the effect of any fertilization regime, since this offers the advantage of strong climate continuity, a longer duration of soil dynamics, and accurate and abundant data. This approach thus deserves more attention^16,17^.

Fertilization could influence the edaphic factors and fungal diversity in soil, consequently adjusting soil fertility, and different fertilization regimes can bring about huge disparities in fungal community diversity. For example, long-term chemical fertilization and organic fertilization of a reclaimed sandy agricultural ecosystem was able to considerably influence its soil physicochemical properties, as well as the abundance and community diversity of soil fungi^18^. In other work, different fertilization regimes (chemical and organic in form) could remarkably change soil properties, such as the soil pH, as well as its fungal community composition^19–22^. Chemical fertilization could increase the abundance of certain phyla, namely Basidiomycota and Chytridiomycota, while organic fertilization may promote the abundance of Ascomycota. Further, adding and mixing organic matter into chemical fertilizer could markedly increased soil fungal community richness^23^. Such phenomena have also been reported to occur in yellow clayey soil and paddy soil from China^24^. Organic-inorganic combined fertilization dramatically increased fungal community diversity and decrease the prevalence of pathogenic fungi in yellow clayey soil compared with the application of inorganic fertilizer. Combining chemical fertilizer with organic manure reportedly increased some soil properties, and remarkably altered the fungal community structure in paddy soil compared with a single application of chemical fertilizer^25^. However, far fewer studies report on the use of long-term fertilizer experiments to analyze the effects of the three main fertilization regimes on soil fungal community diversity of the North China Plain.

In this paper, we sampled fluvo-aquic soil from the Dezhou experimental station (experiment begun in 1986), the most representative soil in North China Plain. Our aim was to investigate the impact of three fertilization regimes—chemical fertilizer, organic manure, and their combination—under equal nitrogen input on soil fungal diversity and community composition. Compared with previous reports, this study it the first to use an equal nitrogen input among all imposed treatments in testing for their effects upon fungal community composition under long-term fertilization in the North China Plain. This research will help to further establish a scientifically based fertilization regime.

## Materials and Methods

### Field site

**Figure 1:**
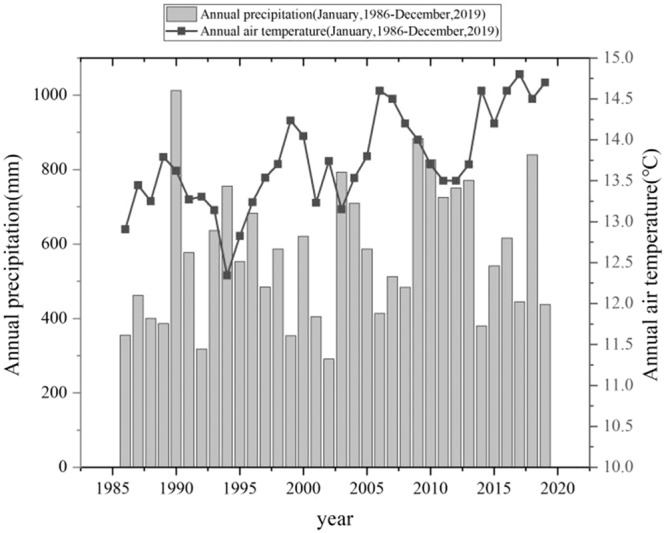
Annual total precipitation (mm) and annual air mean temperature (°C) collected from January 1986 through December 2019, in Yucheng city, China.

The field experiment is managed by the Dezhou experimental station, Chinese Academy of Agricultural Sciences, and located at Yucheng city, in Shandong Province, China (116°34′E; 36°50′N). The environment here is highly suitable for crop growth, having a warm temperate semi-humid monsoon climate; the local water surface evaporation, frost-free season, annual sunshine duration, and mean annual temperature and precipitation values are 2095 mm, 206 days, 2640 h, 13.4 °C and 569.6 mm, respectively (Fig. 1). The long-term field experiment has been running since 1986, using the fluvo-aquic type soil. The baseline soil (0–20 cm layer) had the following characteristics: total organic carbon, 3.93 g kg^−1^; total nitrogen, 0.51 g kg^−1^; alkali-hydrolyzale nitrogen, 37.5 mg kg^−1^; available phosphorus, 17.2 mg kg^−1^; available potassium, 88 mg kg^−1^; and soluble salt, 0.96 g kg^−1^.

### Experimental design and soil sampling

The experiment was conducted on double-cropping system farmland using the rotation of winter wheat and summer maize, four treatments are arranged with four replications (total 16 plots): (1) no chemical fertilizer or organic manure, set as control (CK); (2) conventional chemical fertilization (CF); (3) conventional organic manure fertilization (CM); (4) combined fertilization with chemical fertilizer and organic manure (MF: 1/2 CF + 1/2 CM). The nutrient fertilizer application rates in the chemical fertilization group depended on local farmers’ practice, consisting of nitrogen at 187.5–225 kg ha^−1^, P_2_O_5_ at 112.5–150 kg ha^−1^, and K_2_O 75 kg ha^−1^ per year. Fertilizer used in the organic manure treatment was matured cattle manure; its input was based on total nitrogen content compared with the chemical fertilizer treatment. Each plot was 28 m^2^ in size (4 m × 7 m), with a 0.8-m concrete slab separating the plots.

All the soil was collected in October 2018, by taking 10 random soil cores (6-cm diameter) of 0–20 cm depth layer from each plot (total of 40 samples per treatment); these were mixed to form a composite soil sample per plot. Hence a total of 16 composite soil samples were obtained and stored at –80 °C for the soil properties and fungal community analysis.

### Soil properties analysis

All soil samples were air-dried at room temperature and filtered through a 2-mm mesh sieve for this analysis. Soil pH was measured using a glass combination electrode with a soil-to-water ratio of 1:2.5^26^. Soil organic carbon (SOC) was measured by dichromate oxidization^27^ and total nitrogen (TN) determined by the Kjeldahl digestion method, as previously described^28^. Available potassium (AK) and phosphorus (AP) were detected by a modified resin extraction and the ammonium acetate method, respectively^29,30^. Soil nitrate N (NO_3_^−^) and ammonium N (NH_4_^+^) were extracted by 2 M KCL solution (1:5, w/v) for 30 min, and the concentrations of NO_3_^−^ and NH_4_^+^ were measured using a flow injection auto-analyzer (AA III, Seal Analytical, Norderstedt, Germany)^31^.

### Soil DNA extraction and PCR amplification

Total DNA in each soil sample was extracted by using a FastDNA Spin Kit for soil (MP, Santa Ana, USA), according to the manufacturer’s protocol. The quality and concentration of extracted DNA extracted was checked and detected using a NanoDrop 2000 (Thermo Scientific, Wilmington, USA), and DNA integrity verified by 1% agarose gel electrophoresis.

The primer pairs ITS1F (5′-CTTGGTCATTTAGAGGAAGTAA-3′) and ITS2R (5′-GCTGCGTTCTTCATCGATGC-3′) were used to amplified the ITS regions of the extracted DNAs. The PCR reaction system consisted of 2 μl of 2.5 mM dNTPs, 0.8 μl of ITS1F and ITS2R primers (5 μM), 0.2 μl of rTaq Polymerase, 0.2 μl of BSA, 10 ng of DNA template, 2 μl of buffer, with ddH_2_O added to a final volume of 20 μl. The PCR program was run on a thermocycler PCR system (ABI GeneAmp 9700, Foster, United States) as follows: denaturation at 95 °C for 3 min; 35 cycles at 95 °C for 30 s, annealing at 55 °C for 30 s, and extension at 72 °C for 45 s; ending with an extension at 72 °C for 10 min. All PCR products were detected by 2% agarose gel electrophoresis and recovered by the AxyPrep DNA Gel Extraction Kit (Axygen, Union City, USA).

### Illumina MiSeq sequencing and data processing

The barcoded PCR products were used to construct MiSeq libraries with the TruSeqTM DNA Sample Prep Kit (Illumina, San Diego, USA) according to the manufacturer’s instructions. These libraries were then paired-end (2×300) sequenced on an Illumina MiSeq platform (Illumina, San Diego, USA) by the Majorbio Co. Ltd., Shanghai. All obtained sequences were deposited into the NCBI Sequence Read Archive (SRA) to receive accession numbers.

Raw sequence data were then assembled by the FLASH program and filtered using Trimmomatic software^32,33^. Raw data that contained an N base, or had an average quality score <20 and sequence length <50 bp were discarded.

### Bioinformatic and statistical analysis

Operational taxonomic units (OTUs) with 97% similarity cutoff were clustered using UPARSE software, and their taxonomy analyzed with the RDP classifier using the Unite database, with a 70%-confidence threshold applied.

Several alpha diversity indices—Sobs, Shannon-Weaver, Simpson’s 1-D, ACE, Chao 1, and coverage—were calculated and used to evaluate the richness and diversity of the soil fungal community. The Wilcoxon rank-sum test was used to analyze alpha diversity. Principal coordinate analysis (PCoA) was used to capture and convey significance differences in fungal community compositions among all fertilization regimes. Unweighted unifrac was used to calculate the distance metric and PERMANOVA was used for the statistical analysis. To identify differences and quantify how much fungal abundance changed among the different treatments, the linear discriminant analysis Effect Size (LEfSe) was used with a linear discriminant analysis (LDA) score > 2. The assumptions of a normal distribution and homogeneity of variance of the data were checked, then significant differences among treatments in their effects on soil and fungi response variables were investigated with one-way ANOVAs and Duncan’s multiple range test for post hoc comparisons in SAS v9.1.3 (SAS Institute Inc., Cary, NC, USA), for which a *P* value <0.05 was considered significant.

## Results

### Soil properties after long-term fertilization

Long-term (32 years) fertilization led to stark differences in soil properties under the different fertilization regimes (Table 1). Compared with the control, all three fertilization regimes significantly improved soil AP and NO_3_^−^, but the combination of 1/2 chemical fertilizer and 1/2 organic manure and organic manure treatments significantly reduced soil pH and increased soil TN, SOC, AP, AK, and NO_3_^−^. By contrast, chemical fertilizer treatment was similar to the control in its effects, except upon AP and NO_3_^−^. Among the three fertilization regimes, the combination of 1/2 chemical fertilizer and 1/2 organic manure, as well as organic manure treatments, significantly increased soil TN, SOC, AP and NO_3_^−^ compared with chemical fertilizer treatment; however, soil C:N ratio was negligibly affected by manure inputs. Applying only organic manure significantly improved SOC, AP, AK, and NO_3_^−^, but reduced NH_4_^+^ content in soil when compared with the combined application of chemical and manure fertilizer.

**Table 1:** Soil properties after long-term (32 years) fertilization.

Treatment	pH value	TN	SOC	NH_4_^+^	NO_3_^−^	AP	AK	C:N
CK	8.71 ± 0.06a	0.78 ± 0.13a	6.57 ± 1.21a	6.64 ± 2.20a	1.20 ± 0.39a	4.38 ± 0.77a	65.65 ± 6.93a	8.43 ± 0.38a
MF	8.48 ± 0.08b	1.25 ± 0.12b	10.97 ± 1.10b	11.80 ± 2.42b	10.02 ± 1.98b	103.58 ± 21.79b	78.12 ± 4.10b	9.28 ± 2.01ab
CM	8.42 ± 0.04b	1.59 ± 0.28b	16.07 ± 1.52c	7.07 ± 1.55a	20.84 ± 3.78c	186.10 ± 14.34c	148.41 ± 24.02c	10.25 ± 1.35b
CF	8.58 ± 0.08ab	0.88 ± 0.12a	7.72 ± 0.63a	6.29 ± 2.65a	4.64 ± 1.87d	31.38 ± 2.45d	82.85 ± 12.42ab	8.88 ± 0.54ab

TN: total N (g kg^−1^); SOC: soil organic carbon (g kg^−1^); AP: available P (mg kg^−1^); AK: available K (mg kg^−1^). Different letters in the column represent significant differences (*P* < 0.05) according to Duncan’s multiple range test.

### Fungal α-diversity analysis

Illumina MiSeq sequencing and data processing generated 1, 832, 804 sequences in total from the 16 soil samples, averaging 262 bp in length. All obtained sequences were deposited into SRA under the accession number SRR8942689. All the sequences were clustered into 1, 344 OTUs based on 97% similarities, of which 743, 706, 702, and 731 OTUs were obtained from the control, chemical fertilizer, organic manure, and combination of 1/2 chemical fertilizer and 1/2 organic manure treatment groups, respectively.

**Figure 2:**
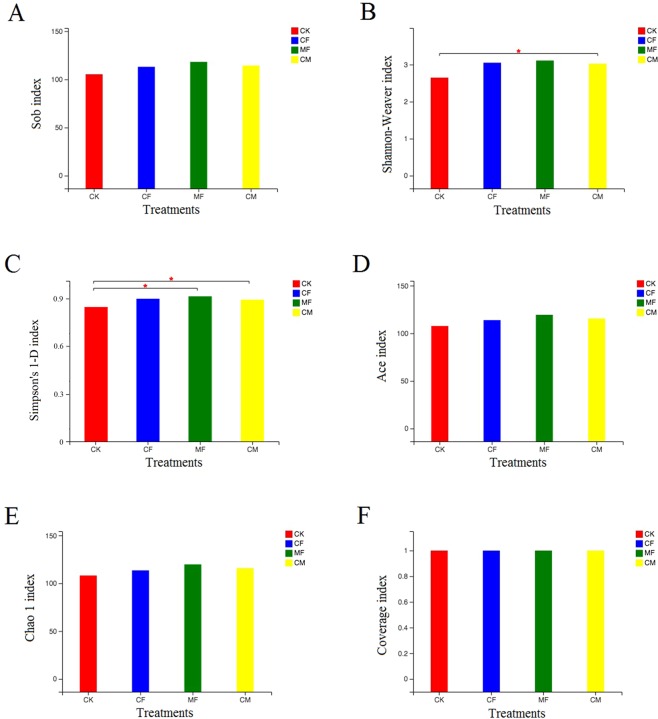
Soil fungal alpha diversity analysis of different fertilization regimes using six indexes. (A) Sobs, (B) Shannon-Weaver, (C) Simpson’s 1-D, (D) ACE, (E) Chao, and (F) Coverage. Statistical differences were analyzed by the Wilcoxon’s rank-sum test. The x-axis represents different fertilization treatments; y-axis indicates the index at the genus level. CK, no chemical fertilizer or organic manure; CF, chemical fertilization; CM, organic manure fertilization; MF, combination of 1/2 CF and 1/2 CM. *P < 0.05.

Six indices were used for evaluating fungal community richness and diversity in soil (Fig. 2). They revealed that fungal diversity differentially changed after long-term fertilization, in that the organic manure treatment group had higher Simpson’s 1-D and Shannon-Weaver indices than the control, indicating that organic manure inputs promoted soil fungal diversity. Yet no significant differences in fungal species richness were detected according to the Sobs, Chao, and ACE indices, indicating similar effects from the fertilization regimes on fungal communities in this soil.

### Fungal β-diversity analysis

**Figure 3:**
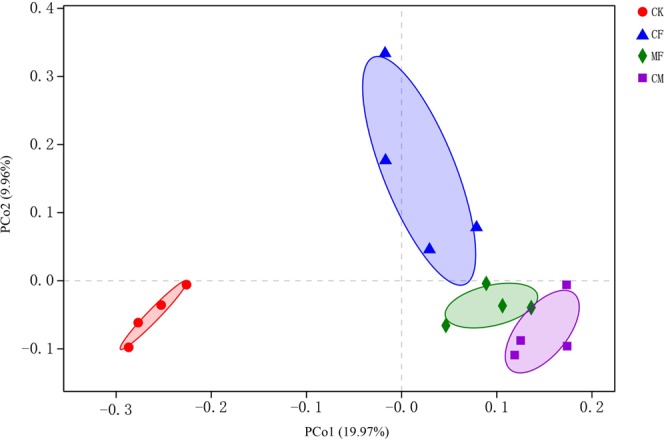
Principal coordinates analysis (PCoA) of soil fungal communities at the OTU level among different fertilization regimes. The x- and y-axis each represent variance in soil fungal community structures. CK, no chemical fertilizer or organic manure; CF, chemical fertilization; CM, organic manure fertilization; MF, combination of 1/2 CF and 1/2 CM.

PCoA was used to analyze the fungal community structure of each treatment. The PERMANOVA had a P-value of 0.001, which demonstrated that a significance difference existed among different treatments. The principal coordinates 1 (PCo1 axis) and 2 (PCo2 axis) respectively explained 19.97% and 9.96% of the variance in soil fungal community structures (Fig. 3). According to this PCoA ordination, control plots located to the left, clearly separated from the chemical fertilizer, organic manure, and combination of 1/2 chemical fertilizer and 1/2 organic manure plots which clustered to the right; this indicated fungal community structure had changed significantly after fertilization. Nonetheless, organic manure and the combination of 1/2 chemical fertilizer and 1/2 organic manure plots clustered together to the right bottom part of the ordination space, which suggested their soils harbored more similar fungal community structures. Conversely, chemical fertilizer plots were located in the right upper part, well separated from organic manure and the combination of 1/2 chemical fertilizer and 1/2 organic manure treatment groups, thus indicating the adding of manure to soil could change its fungal community structure.

### Fungal community composition

**Figure 4:**
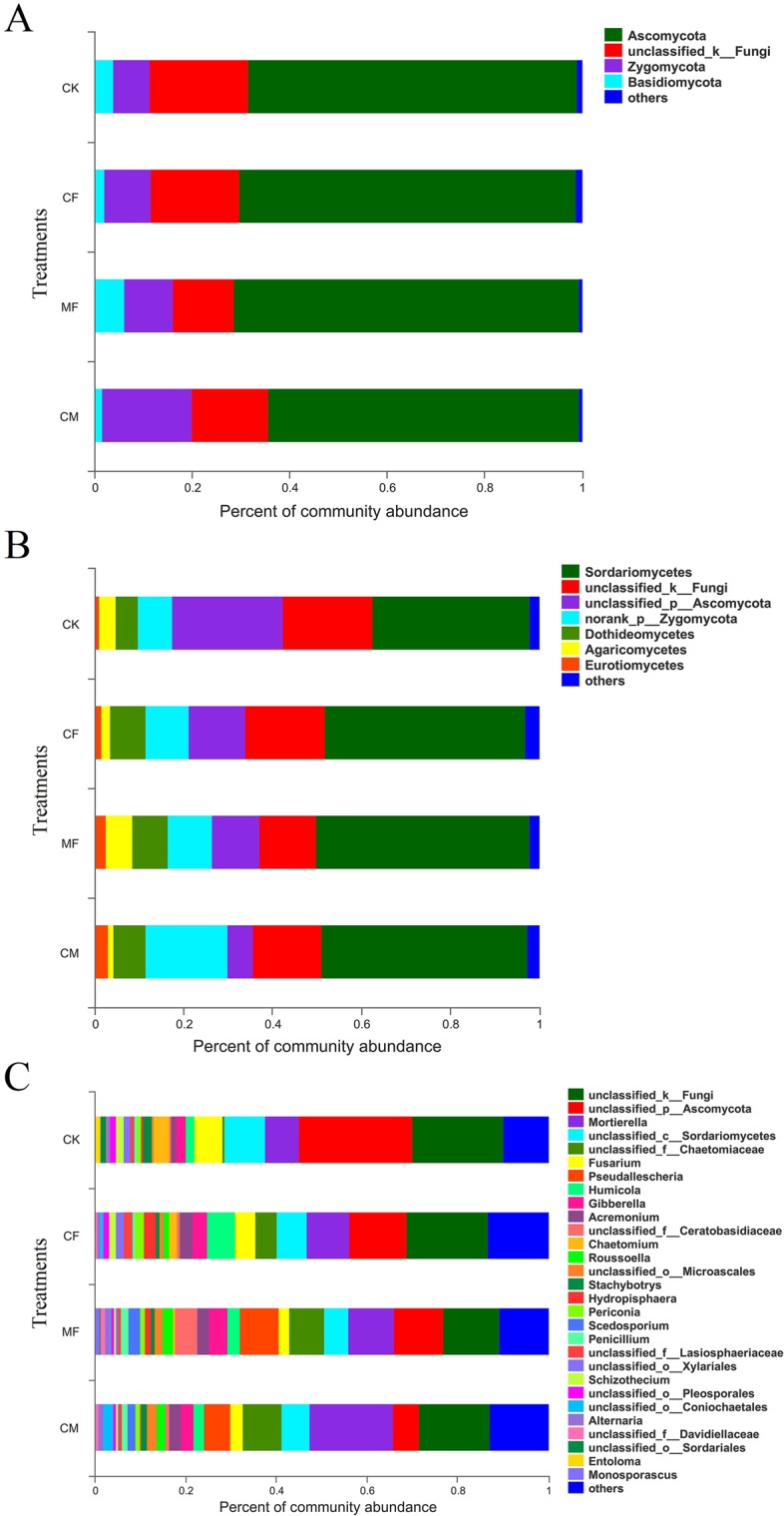
Soil fungal community composite analysis under three taxon levels among different fertilization regimes. (**A**) Phylum level, (**B**) Class level, (**C**) Genus level. X-axis represents the abundance of fungal community composite; Y-axis means different fertilization treatments. CK, no chemical fertilizer or organic manure; CF, chemical fertilization; CM, organic manure fertilization; MF, combined 1/2 CF and 1/2 CM.

The phylum taxa (>1%) were similar across the four treatments, in being dominated by Ascomycota (64.0–70.8%), followed by unclassified_k_fungi (12.6–20.5%), Zygomycota (7.6–18.5%), and Basidiomycota (1.6–6.0%). Apparently, none of the fertilization regimes affected the fungal community composition at the phylum level, but the abundance of Zygomycota in the organic manure treatment was higher than the other three groups (Fig. 4A). A similar situation was evident at the taxon level of class, in that the same seven dominant classes appeared: Sordariomycetes (35.4–48.4%), unclassified_k_fungi (12.6–20.1%), unclassified_p_Ascomycota (5.7–25.1%), norank_p_Zygomycota (7.6–18.4%), Dothideomycetes (5.1–7.9%), Agaricomycetes (1.3–5.9%), and Eurotiomycetes (0.9–3.1%). However, the abundance of Sordariomycetes was higher in the three fertilization regimes compared with the control (Fig. 4B).

Fungal community composition showed conspicuous significant differences among treatments when examined at the genus level. In addition, the dominant fungal genera also differed (Fig. 4C) among them as follows: in the control soil, *Ascomycota* (25.1%) was most dominant, followed by unclassified_k_fungi (20.1%), *Sordariomycetes* (8.77%), *Mortierella* (7.5%), and *Fusarium* (6.1%). In chemical fertilizer treatment soil, the dominant genus was instead unclassified_k_fungi (18%), followed by *Ascomycota* (12.7%), *Mortierella* (9.4%), *Sordariomycetes* (6.5%), and *Humicola* (6.2%). Under the organic manure treatment, however, the dominant genus was *Mortierella* (18.4%), then unclassified_k_fungi (15.6%), *Chaetomiaceae* (8.7%), *Sordariomycetes* (6.1%), and *Pseudallescheria* (5.9%). For the combination of 1/2 chemical fertilizer and 1/2 organic manure treatment soil, its dominant genus was unclassified_k_fungi (12.6%), not unlike under the chemical fertilizer treatment, followed by *Ascomycota* (10.7%), *Mortierella* (10.1%), *Pseudallescheria* (8.6%), and *Chaetomiaceae* (7.7%). Hence, fertilization dramatically influenced both fungal community composition and abundance compared with the control, but this effect depended on the fertilization regime applied. Moreover, adding organic manure to soil was found to increase the abundance of *Penicillium*, *Pseudallescheria*, *Microascales*, and *Scedosporium*.

**Figure 5:**
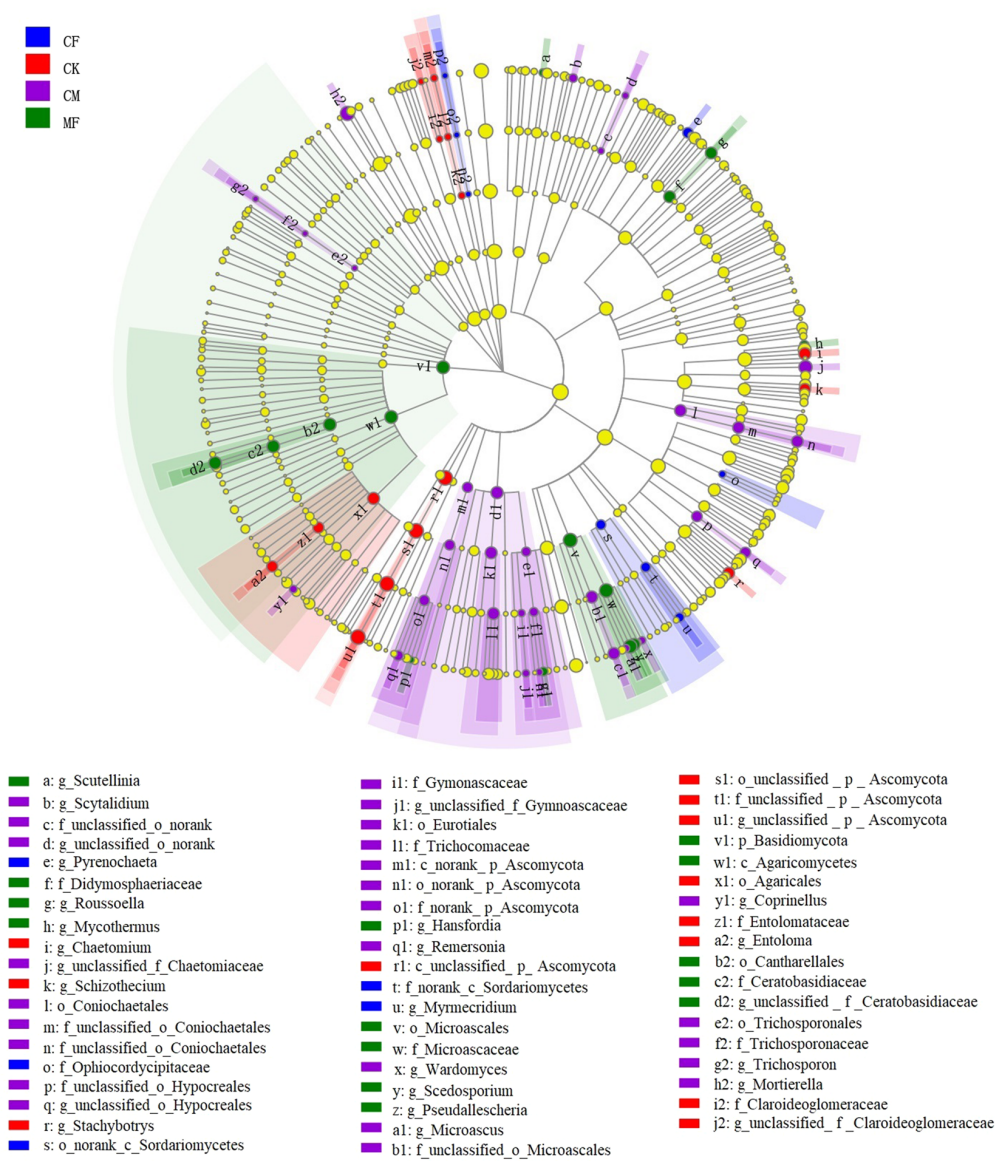
Linear discriminant analysis Effect Size (LEfSe) analysis of a taxonomic cladogram at five taxon levels among different fertilization regimes. Circles from the inside to outside of the cladogram represent fungal community at phylum, class, order, family and genus levels. Red, blue, green, and purple coloring represent the four fertilization treatments; yellow circles at each taxon level denotes a fungal community not significantly different among the four fertilization regimes. CK, no chemical fertilizer or organic manure; CF, chemical fertilization; CM, organic manure fertilization; MF, combination of 1/2 CF and 1/2 CM.

The LEfSe analysis (based on an LDA score > 2) detected 1 phylum, 4 classes, 12 orders, 17 families, and 32 genera in all treatments (Fig. 5). Fungal composition at the genus level differed among the four treatments, in that the control contained 9 genera (unclassified_p_Ascomycota had the highest LDA score); chemical fertilizer treatment contained just 3 genera, with *Pyrenochaeta* receiving the highest LDA score; organic manure treatment contained the most genera, with 12, of which *Mortierella* had the highest LDA score, and: combination of 1/2 chemical fertilizer and 1/2 organic manure treatments had 8 genera with the highest LDA score assigned to *Pseudallescheria*.

## Discussion

Soil fungal alpha and beta diversity was significantly altered by the three main fertilization regimes. Compared with the control, the application of organic manure strongly shaped soil fungal diversity, a result consistent with previously reported findings^34–37^. In our study, the Shannon-Weaver and Simpson’s 1-D indexes of fungal alpha diversity were significantly affected by applying organic manure and the combination of 1/2 chemical fertilizer and 1/2 organic manure treatments to soil (Fig. 2). This effect could be due to the organic manure, since it can improve the quantity of SOC, which is necessary for local fungal growth. Additionally, organic manure contains abundant nutrients, which could promote fungi growth, leading to the marked improvements in soil fungal diversity found after organic manure fertilization.

However, not all long-term fertilization regimes were able to significantly alter soil fungal diversity. The chemical fertilizer alone also influenced on soil fungal diversity when compared with the control, but the difference between them in this effect was not significant (Fig. 2). This result agrees with Yao^38^, who found that fertilization with chemical fertilizer had a negligible influence on fungal diversity in soils of a degraded arid steppe, and this phenomenon was reported for acid tea-garden soil^39^. The most plausible explanation for this trivial effect from chemical fertilization is that only slightly changes soil basic properties, especially its pH value and SOC that are known to be essential for higher fungal diversity^40^. We found neither markedly changed by the chemical fertilizer treatment, further suggesting fungal diversity in fluvo-aquic soil was not affected by chemical fertilization. Beta diversity analysis showed that fertilization could remarkably influence the fungal community composition compared with control, which could be explained by the extra carbon and nitrogen sources supplemented by chemical fertilizer and organic manure. Compared with single chemical fertilization, single organic manure fertilization and the combination of 1/2 chemical fertilizer and 1/2 organic manure fertilization induced a more similar fungal community composition, perhaps because of the higher SOC content in the organic manure treatment. The SOC content has been reported as the dominant factor in determining the fungal community composition^41,42^.

Different fertilization regimes played vital roles in shaping soil fungal community composition. At the phylum level, this composition was similar among the three fertilization regimes, with fungal communities dominated by Ascomycota, unclassified_k_Fungi, Zygomycota and Basidiomycota. That Ascomycota was most dominant in all fertilization regimes is consistent with other reports, which indicated that fluvo-aquic soil of North China Plain is favorable for Ascomycota growth. However, the abundance of unclassified_k_Fungi was lower in the organic manure and combination of 1/2 chemical fertilizer and 1/2 organic manure treatments compared with the control and chemical fertilizer treatment, probably because organic manure fostered the population growth of other phyla capable of using it a substrate for propagation.

At the class level, fungal community composition among all the fertilization regimes was mostly uniform. Although the most dominant class in each was Sordariomycetes, the abundance of Sordariomycetes in the chemical fertilizer, organic manure, and combination of 1/2 chemical fertilizer and 1/2 organic manure treatments exceeded the control, perhaps because of sufficient nutrition from fertilizer beneficial for Sordariomycetes growth^43^. In addition, Eurotiomycetes were more abundant in the organic manure and combination of 1/2 chemical fertilizer and 1/2 organic manure treatments than in either the control or chemical fertilizer treatment, and its abundance was correlated with N concentration, suggesting that higher N in soil may favor Eurotiomycetes^44^. This interpretation is supported by the fact total N content in manure-fertilized soil was higher than in chemical fertilization, as well as no fertilization.

At the genus level, dominance was not similar among the fertilization regimes. *Fusarium* is the fungal pathogen most responsible for causing major damage to wheat and maize production^45–48^. Its abundance in the control and chemical fertilizer treatment was higher than in organic manure and combination of 1/2 chemical fertilizer and 1/2 organic manure treatments, suggesting that manure applications could reduce the risk of crop disease caused by *Fusarium*. Compared with the control and chemical fertilizer treatment, *Penicillium* was more abundant in both organic manure and chemical fertilizer treatments; this genus can promote the growth of wheat and maize plants, and improve the environmental stress tolerance of both crops^49–51^. Our results imply organic manure fertilization possesses the potential to enhance the growth and stress tolerance of these two staple crops.

Soil fungal community composition and diversity was found to be closely related to soil properties, which in turn are altered by different fertilization regimes^52–54^. Among these properties, the soil pH value is a crucial factor^5,24^. Commonly, nitrogen fertilizer retains hydrogen (H^+^) into the soil, which could lead to a decreased pH value in soil^55–57^. However, in our long-term experiment, the chemical fertilization N, P, and K elements were balanced inputs, so no excess hydrogen was stored in the experimental plots’ soil^14,58,59^. Evidence for the lower soil pH value phenomenon was found under the organic manure fertilization regimes, as chemical fertilizer and combination of 1/2 chemical fertilizer and 1/2 organic manure treatments had lower pH values than control. This could be explained if organic manure promoted the abundance of fungi that can produce organic acids. The genus *Mortierella*, which is capable of acid production, was found more abundant under organic manure fertilization than in the control, and so it might have driven the pH value lower in plots with organic manure added^60,61^.

SOC is another crucial property of soil that could influence its fungal community composition and diversity^54,62,63^. Higher SOC content in soil could provide enough carbon sources for soil microbial’ growth, thereby influencing microbial community composition and diversity under higher SOC inputs^64^. The SOC content under chemical fertilization was unchanged relative control soil, but it was considerably enhanced by manure fertilization (organic manure and combination of 1/2 chemical fertilizer and 1/2 organic manure treatments); their improved SOC contents over the other treatments arose from the manure component added. Due to higher SOC in organic manure fertilization, its fungal community composition and diversity was significantly altered compared with that of the chemical fertilizer or control. Moreover, fungi such as *Penicillium* could increase the biomass of crops. If so, their crop residues would have been greater than those under control and chemical fertilizer treatments, which is another argument for the enhanced SOC content^65,66^.

Excessive application of phosphorus risks environmental pollution. Reportedly, the threshold concentration for phosphorus application is 30 mg kg^−1^; when this is exceeded in soil, there is an augmented risk of phosphorus entering groundwater via soil runoff and drainage, resulting in phosphorus pollution^67^. In our study, all the fertilization regimes led to significantly increased available phosphorus contents, but the single application of organic manure generated the highest value, of 186.1 mg kg^−1^, more than 6 times the threshold concentration above. Hence, single organic manure fertilization greatly elevates the risk of phosphorus pollution in fluvo-aquic soil. Both organic manure and combination of 1/2 chemical fertilizer and 1/2 organic manure treatments exhibited similar fungal community composite and diversity, but the available phosphorus content in the combination of 1/2 chemical fertilizer and 1/2 organic manure treatment was significantly lower than in the organic manure treatment. For this reason, we recommend the combined 1/2 chemical fertilizer and 1/2 organic manure fertilization, coupled to reductions in phosphorus content in chemical fertilizers, as an ideal regime in future agricultural applications.

In conclusion, on the North China Plain, long-term (32-years) fertilization significantly altered key soil properties, as well as soil fungal diversity and community composition, under different fertilization regimes. In particular, the use of organic manure treatments (i.e., the organic manure and combination of 1/2 chemical fertilizer and 1/2 organic manure treatments) was able to dramatically influence soil properties, namely by increasing TN, SOC, AP and NO_3_^−^ (compared with the control). Furthermore, applying organic manure remarkably enhanced soil fungal diversity and community composition compared with the control, an effect that chemical fertilization could not achieve. Finally, compared with the control and chemical fertilizer treatment, applying organic manure reduced the abundance of soil-born fungal pathogens such as *Fusarium*, suggesting its use (as organic manure alone or in the treatment combination of 1/2 chemical fertilizer and 1/2 organic manure) could impair soil-born fungal diseases.

## Data Availability

All obtained sequences data were deposited into SRA under the accession number SRR8942689.
